# Eugenol transport and biosynthesis through grafting in aromatic plants of the *Ocimum* genus

**DOI:** 10.5511/plantbiotechnology.24.0124a

**Published:** 2024-06-25

**Authors:** Shogo Hirose, Kaito Sakai, Sawa Kobayashi, Masato Tsuro, Atsushi Morikami, Hironaka Tsukagoshi

**Affiliations:** 1 Faculty of Agriculture, Meijo University

**Keywords:** eugenol, eugenol synthases, grafting, *Ocimum basilicum*

## Abstract

Aromatic compounds play essential roles in plant physiology and various industries because of their unique fragrances and beneficial properties. In this study, we investigated the transport and biosynthesis of eugenol, a prominent aromatic compound, within the *Ocimum* genus, using grafting experiments. Grafting sweet basil (*Ocimum basilicum*) scions onto diverse rootstocks, including tobacco (*Nicotiana benthamiana*) and thyme (*Thymus vulgaris*), revealed that eugenol is transported from the shoot to the root across distinct plant species. Furthermore, grafting within the *Ocimum* genus, which includes *O. basilicum*, *O. tenuiflorum*, and *O. americanum*, resulted in variations in eugenol transport and accumulation. The eugenol content in the shoots remained constant across all combinations, whereas the root eugenol levels varied depending on the scion-rootstock pair. To elucidate the biosynthetic capabilities of eugenol in *Ocimum* roots, we performed in vitro enzyme assays using crude protein extracts from roots, which revealed that eugenol can be synthesized in roots in addition to being transported. Expression analysis of eugenol synthase (EGSs) genes showed that *EGS4* expression was influenced by grafting in *O. basilicum* roots, suggesting compensation by other EGSs. Our results suggest that eugenol transport and biosynthesis are multifaceted processes influenced by the interactions between different species and tissues. The potential to engineer eugenol levels in rootstocks lacking biosynthetic capacity has potential applications in agriculture and industry. This study reveals the dynamic interplay between eugenol transport and biosynthesis in the *Ocimum* genus, providing insights into the manipulation of aromatic compound production in plants.

## Introduction

Aromatic plants, including herbs, exhibit fragrances unique to each species. Fragrance, characterized by a volatile essence referred to as aroma, becomes perceptible when these aromatic compounds volatilize and disperse in the air. Fragrance is defined by the relative proportions of a diverse array of aromatic constituents. The current number of recognized plant species ranges from 200,000 to 300,000. Conversely, data from a repository encompassing 100,000 plant species estimates the total metabolites produced by plants to be approximately one million (Afendi et al. 2012). Among these metabolites, a subset of over 80,000 compounds, notably terpenoids, which are renowned for their aromatic qualities, have been identified (Christianson 2018). These aromatic compounds have extensive utility across multiple sectors, functioning as additives in the food industry, contributing to medicinal applications such as aromatherapy (Kuriyama et al. 2005), and serving as pharmacological agents that confer anti-allergenic properties (Ito et al. 1998). Consequently, the use of aromatic compounds is of paramount importance in a diverse spectrum of industries.

Terpenoids and phenylpropanoids are the major aromatic compounds produced by plants. Terpenoids are biosynthesized via isoprenoid pathways, such as the cytoplasmic mevalonate and chloroplast 2-*C*-methyl-D-erythritol-4-phosphate pathways (Fock-Bastide et al. 2014; Karunanithi and Zerbe 2019; Koeduka et al. 2009, 2006; Vassao et al. 2006). The aromatic compounds in isoprenoids include alpha-pinene and limonene. Phenylpropanoids biosynthesis begins with the aromatic amino acid, phenylalanine. These phenylpropanoids, which are biosynthesized by plants, include eugenol, chavicol, and isoeugenol as aromatic compounds (Louie et al. 2007). Plants use these aromatic compounds in diverse roles, including defense against feeding organisms and microorganisms, signaling to other individuals, and attracting pollinators (Dudareva and Pichersky 2000; Schmelz et al. 2014). Because of the importance of these aroma compounds, controlling their production in plants will provide many advantages for plant engineering.

Plants have a sessile lifestyle, which means that they communicate within individual plants, such as between roots and shoots, through the transport of proteins and chemicals in response to environmental changes. Strigolactone, a plant hormone, is transported from roots to shoots and regulates axillary bud development (Domagalska and Leyser 2011). Similarly, during nitrogen metabolism, the C-terminally encoded peptide hormone, is transported from the roots to the shoots, transmitting nitrogen deficiency signals throughout the plant (Okamoto et al. 2016). In addition to growth- and development-regulating plant hormones, the transport of secondary metabolites has also been uncovered. Grafting is a valuable tool for investigating the movement of these compounds between plant organs. Despite its longstanding use in agriculture, the molecular mechanisms underlying graft establishment have remained elusive until recently (Notaguchi et al. 2020). As a result, the successful grafting of diverse plant species has been achieved. Thus, grafting can be used to reveal the movement of unknown compounds within the plant body. If the substrates for the final metabolic compounds synthesized in the shoots and roots are transported through the plant body, it will open the door to engineering, and processes such as the synthesis of novel compounds via grafting between different species can be performed.

In this study, grafting was employed to investigate changes in the aromatic compounds of herbal plants. We focused on sweet basil, *Ocimum basilicum*, because of its well-documented composition of aromatic components (Koeduka et al. 2006) and reported mRNA transcriptome data (Dhar et al. 2020). In sweet basil, eugenol is predominantly biosynthesized in the peltate glandular trichomes (PGTs) of the leaves and is thought to be transported from the shoot to the root in camphor basil, *Ocimum kilimandscharicum* (Singh et al. 2020). Our grafting experiments revealed the transport of eugenol from shoots to roots, even across distinct plant species, such as basil, tobacco, and thyme. Furthermore, we grafted the *Ocimum* genus, which revealed not only the transportation but also the synthesis of eugenol in the roots. To assess eugenol biosynthetic activity in *Ocimum* roots, we conducted an in vitro enzyme assay using crude protein extracts obtained from these roots. The results of this enzyme assay indicated the presence of eugenol biosynthetic activity in crude protein extracts from various species of the genus *Ocimum*. Expression analysis showed that the expression of *eugenol synthase 4* (*EGS4*) in sweet basil roots is influenced by grafting. However, other eugenol synthase genes (*EGS*s), such as *EGS3*, *EGS6*, and *EGS7*, are expressed at the same level as in the non-grafted sweet basil. Therefore, we hypothesized that the compensation of expression within *EGS* genes occurs through grafting. Remarkably, basil exhibits the ability to transport eugenol from the shoot to the root, even when grafted across different plant species. Our findings propose the intriguing possibility of employing basil as a scion to manipulate eugenol levels in rootstocks that inherently lack eugenol biosynthesis capability. Our results provide an example of how grafting can provide a novel biological role for chemical transport between different plant species.

## Materials and methods

### Plant materials and growth conditions

Commercially available sweet basil (*Ocimum basilicum*), holy basil (*Ocimum tenuiflorum*), lime basil (*Ocimum americanum*), and thyme (*Thymus vulgaris*) seeds were sterilized using 50% hypochlorite and 0.05% Triton X-100 for 30 min and washed three times with sterilized water, then incubated seeds at 4°C for 1 day. Seeds were germinated on a medium containing 2,000×diluted Hyponex (Hyponex Japan, Osaka, Japan), 0.5% sucrose, and 2% agar, without pH adjustment.

*N. benthamiana* seeds were sterilized using 5% hypochlorite and 0.05% Triton X-100 for 5 min and washed three times with sterilized water, incubated at 4°C for 3 day. Seeds were germinated in a medium containing 1/2×Murashige and Skoog (MS; FUJIFILM Wako Pure Chemical Industries, Ltd., Osaka, Japan), 2% agarose medium supplemented with 0.05% 2-morpholinoethanesulfonic acid monohydrate (MES), and 0.5% sucrose (pH 5.7).

Plants were grown aseptically in a chamber (Panasonic, Osaka, Japan) at 22°C with a 16-h light/8-h dark cycle until reaching approximately 0.1 cm of a hypocotyl width. The composition of the medium was made up of 2,000×diluted Hyponex, 0.5% sucrose, and 2% agarose, without pH adjustment.

### Grafting

After sowing on the medium, basil plants (10-d old) were subjected to scion and rootstock obtained by cutting the middle of the hypocotyl. Thyme and *N. benthamiana* were grown for approximately 20 day on the medium, and the middle of the hypocotyl of these plants was cut to obtain a rootstock. Thyme and tobacco stems were trimmed to approximately 2 cm below the shoot apex to remove any true leaves that could hinder the grafting process.

Grafting was performed by uniting the scion and the rootstock to ensure a firm fit between the cut surfaces. The graft site was enveloped in silicone tubes with a notch for 10 day. Grafting was performed under aseptic conditions using the following medium:

For heterologous grafting with thyme and basil, a 1/2×MS agarose medium supplemented with 0.05% MES at pH 5.7 was used. Cellulase Onozuka R-10 (Yakult Pharmaceutical Industry Co. Ltd., Tokyo, Japan) 0.1% was also added to the medium to enhance plant adhesion, following the method of Kawakatsu et al. (2020). After grafting, the grafted plants were incubated in a chamber at 25°C with a 16-h light/8-h dark cycle for 10 day.

For grafting within the *Ocimum* genus, a 1/2×MS agarose medium was used, supplemented with 0.05% MES, and 0.5% sucrose (pH 5.7), following the method of Tsutsui et al. (2020). After grafting, grafted plants were incubated in a chamber at 27°C with a 16-h light/8-h dark cycle for 10 day on the medium.

After 10 day on the agarose medium, the grafted plants were transferred to vermiculite and grown under continuous light conditions at 22°C. Grafted plants that survived for 4 weeks in the vermiculite were considered successful grafts. As an experimental control, non-grafted plants were grown under the same conditions as those of grafted plants.

### Essential oil extraction

Approximately 0.3 g of young leaves or roots from each of the grafted and non-grafted plants were collected and flash-frozen in liquid nitrogen.

For basil and thyme grafting, plant samples were subjected to extraction of essential oil with 1 ml *n*-hexane supplemented with 100 µg of tetradecane as the internal standard. The extraction process was performed using steam distillation and heating at 180°C for 2 h. After essential oil extraction, only the hexane phase was isolated, and any residual water in the hexane phase was eliminated utilizing 0.3 g of Na_2_SO_4_.

For basil and basil grafting, and basil and tobacco grafting, plant samples were frozen in liquid nitrogen and pulverized. The pulverized samples were combined with 1 ml *n*-hexane and 100 µg of tetradecane as the internal standard. The samples were then agitated at 140 rpm for 2 h. The agitated samples were then centrifugation at 10,000×g for 10 min, and the supernatant was obtained as the extracted essential oil. Three or more biological replicates were used for each experiment.

### In vitro enzyme assay

Approximately 0.3 g of young leaves or roots of sweet, holy, and lime basils were collected and frozen in liquid nitrogen. The plant samples were then pulverized, and 3 ml of protein extraction buffer (50 mM Tris-HCl, pH 8.0, 10 mM NaCl, 10 mM β-mercaptoethanol, and 10% glycerol) was added to 0.3 g of the plant sample, ensuring a mass percentage concentration of 10 : 1 (w/v). The resulting mixture was maintained on ice for 30 min, subsequently centrifuged at 14,000×g, and 4°C for 20 min. The supernatant was collected as the crude protein extract.

For the removal of volatile components such as eugenol from the crude protein extracts, 100 to 400 µl of crude protein extracts were incubated at 37°C for 3 h. After incubation, 1 ml *n*-hexane was added to the crude protein extract. The hexane phase was discarded and the process was repeated three times.

An enzymatic reaction solution was prepared as described by Dhar et al. (2020). The enzyme reaction solution consisted of 100 µl crude protein solution, 50 mM MES-KOH buffer (pH 6.5), 1 mM NADPH, 140 µM acetyl-CoA (Sigma-Aldrich, St. Louis, MO, USA), and 1 mM coniferyl alcohol (Sigma-Aldrich) as a substrate. The solution was incubated at 36°C for 180 min for the enzymatic reaction. After the incubation, 100 µl *n*-hexane and 10 µg tetradecane were added to the enzyme reaction solution as the internal standard. The hexane phase was extracted for GC analysis. Three independent biological replicates were used for each experiment.

### GC-MS analysis

Gas chromatography-mass spectrometry analysis of essential oils from plants and enzyme assay extracts was conducted using a GCMS-QP2010 SE (Shimadzu, Kyoto, Japan). A quantity of 2 µl of samples were injected on Inert Cap5 MS/Sil column (i.d. 0.25 mm×length 30 m×film thickness 0.25 µm, GL Science, Tokyo, Japan). Helium was used as the carrier gas, and the column inlet pressure was set to 100 kPa. The splitting ratio was 1 : 20 (w/w). The sample inlet and detector temperatures were set to 240°C and 270°C, respectively. Mass spectra were recorded at the ionization energy of 70 eV. The oven temperature for each sample was programmed as follows.

For basil and basil grafting among *O. basilicum*, *O. tenuiflorum*, *O. americanum*, basil and *T. vulgaris* grafting, and basil and *N. benthamiana* grafting extracts, the oven was held at 60°C for 5 min during essential oil injection, then increased to 230°C at a rate of 5°C min^−1^, followed by an increase of 30°C min^−1^ to 280°C, and then held for 15 min, resulting in a total run time of 55.67 min.

For enzyme assays extracts, the oven was held at 60°C for 20 min during essential oil injection, then the temperature was increased at a rate of 5°C min^−1^ to 140°C, held for 5 min, followed by an increase of 15°C min^−1^ to 230°C, then an increase of 30°C min^−1^ to 280°C and held at 280°C for 5 min, resulting in a total run time of 53.67 min.

The essential oil components of each sample were identified by comparing their mass spectra with those in the NIST05 mass spectral database. Eugenol was identified by comparing its mass spectrum to that of an authentic compound (FUJIFILM Wako Pure Chemical Industries, Ltd.).

### GC analysis

Quantification of the essential oil components including eugenol from plants and enzyme assay extracts, was performed using gas chromatography (GC-2010 Plus; Shimadzu) equipped with a flame ionization detector (FID). A quantity of 2 µl of samples were injected into the TC-5 column (internal diameter, 0.25 mm, length 30 m; film thickness, 0.25 µm; GL Science). Helium was used as the carrier gas and the column inlet pressure was set to 127.3 kPa. The split ratio of the GC was set to 1 : 10. The oven was ramped up by 5°C min^−1^ to 140°C, held at 140°C for 5 min, then increased by 15°C min^−1^ to 230°C, followed by an increase of 30°C min^−1^ to 280°C, and finally held at 280°C for 5 min, for a total of 53.67 min. The sample inlet and detector temperatures were set to 240°C and 280°C, respectively. The quantity of essential oil components containing eugenol was obtained by standardizing the FID peak area percentage and comparing it with a standard curve.

### RT-qPCR

Total RNA was isolated from 50 to 100 mg of leaves and roots of grafted and non-grafted basil plants using the RNeasy® Mini Kit (Qiagen, Venlo, Netherlands). The extracted RNA was then reverse transcribed into complementary DNA (cDNA) using the ReverTra Ace® qPCR RT Master Mix with gDNA Remover (Toyobo, Osaka, Japan). The RT-qPCR was performed using the THUNDERBIRD® SYBR qPCR Mix (Toyobo) on a real-time PCR Eco system (PCRmax). The primers used in this study are listed in Supplementary Table S1. The RT-qPCR efficiency and CT values were determined using standard curves for each primer set. The efficiency-corrected transcript values of the four biological replicates for all samples were used to determine relative expression values. Each value was normalized to the level of β-*actin* (Rastogi et al. 2014).

### Statistical analyses

All statistical analyses were performed using Microsoft Excel or R software. Details of the analyses are provided in the figure legends.

## Results

### Eugenol moves from shoot scion to root stock

**Figure 1:**
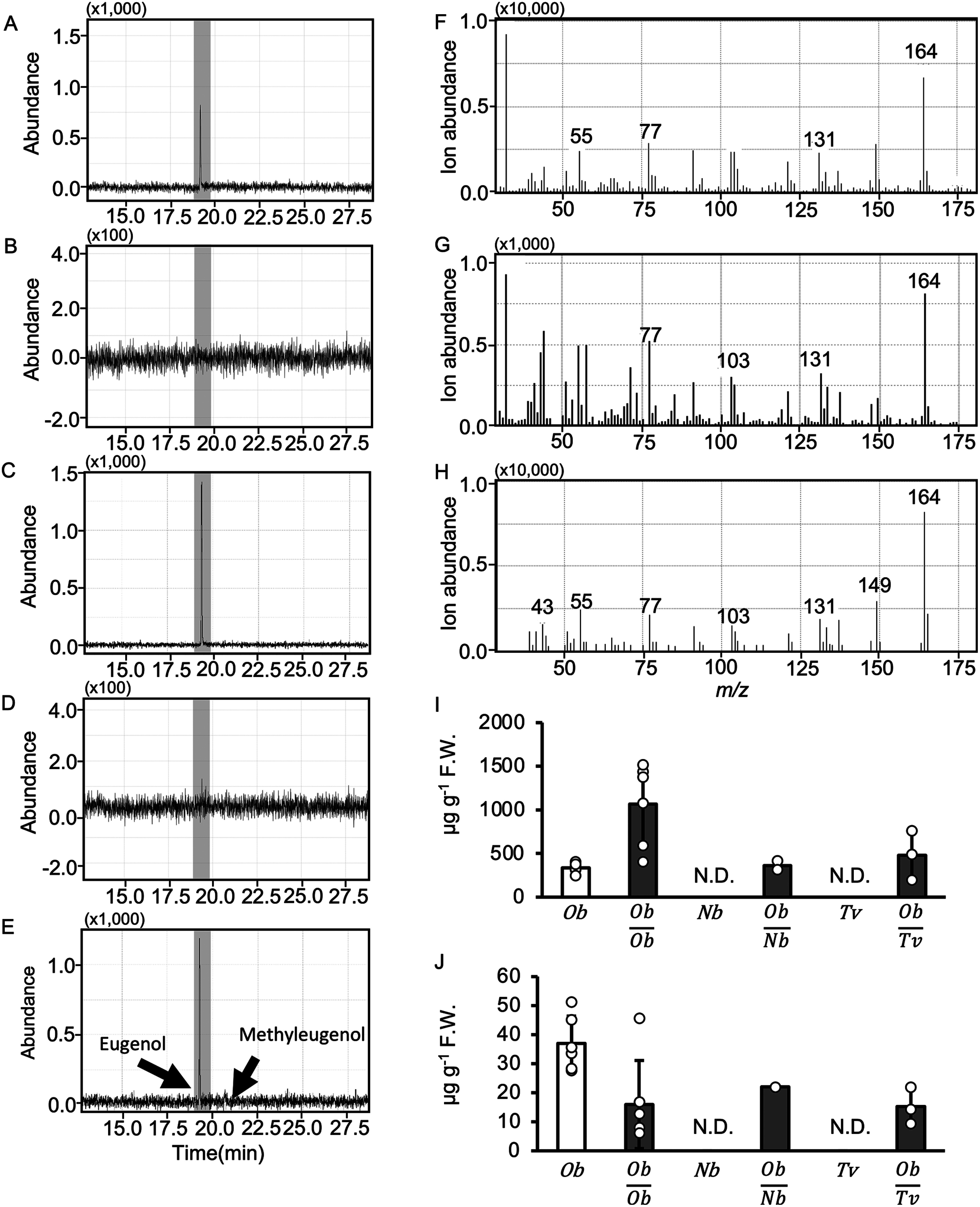
Figure 1. Movement of eugenol from shoot to root across different plant species. Eugenol content in sweet basil and thyme rootstock grafts. (A) to (E) Mass chromatograms of *m*/*z*=164 obtained using GC-MS. (A) Essential oils from sweet basil and tobacco grafted onto roots. (B) Essential oils from tobacco roots. (C) Essential oils from sweet basil and thyme grafted onto roots. (D) Essential oils from thyme root. (E) Eugenol and methyleugenol standards. (A) to (E) Three biological replicates were prepared. (F) and (G) Mass spectra of the reaction products obtained from the GC peaks at a retention time of 19.385 min for (A) and (C), respectively. (H) Fragment pattern of eugenol recorded in the Wiley Library (11th edition). Eugenol content in the shoots (I) and the roots (J) of non-grafted sweet basil, grafted sweet basil scion and sweet basil rootstock, sweet basil scion and tobacco rootstock, non-grafted tobacco, sweet basil scion and thyme rootstock, and non-grafted thyme. Error bars represent±SD (The number of individual plants measured were *Ob*=5, *Ob*/*Ob*=5, *Nb*=3, *Ob*/*Nb*=2, *Tv*=3, and *Ob*/*Tv*=3.). White boxes: non-grafted. Gray boxes: grafted. *Ob*; *O. basilicum*. *Nb*; *N. benthamiana*. *Tv*; *T. vulgaris*. N.D. No detection.

To explore alterations in aromatic compounds caused by grafting between distinct plant species, we conducted grafting experiments on sweet basil (*O. basilicum*), tobacco (*N. benthamiana*), and thyme (*T. vulgaris*). *N*. *benthamiana* was selected as the control because of its high efficiency in interspecies grafting (Notaguchi et al. 2020). Thyme was selected as it belongs to the Lamiaceae family as basil, and the success of grafting within the same family has been indicated in several studies (Goldschmidt 2014; Melnyk 2017). Four weeks post-grafting, we extracted essential oils from both the true leaves and roots of grafted and non-grafted plants and subsequently subjected them to GC-MS analysis. The GC-MS results revealed the presence of distinct eugenol peaks in both tobacco and thyme (Figure 1A, C). In contrast, essential oils extracted from non-grafted tobacco and thyme roots did not exhibit these eugenol peaks (Figure 1B, D). Furthermore, the mass spectra of these eugenol peaks matched those of the control eugenol (Figure 1F, G, H). No new compounds other than eugenol were found in the roots of tobacco and thyme grafted with sweet basil as the scion. The shoot and root essential oil components of *O*. *basilicum* were analyzed using GC. The results showed that eugenol was the primary component of *O*. *basilicum* essential oil in both shoots and roots (Supplementary Table S2). This result is consistent with a previous report showing that eugenol is a well-recognized characteristic compound of the *Ocimum* genus (Anand et al. 2016). Our results suggested that eugenol is transported from the sweet basil scion to the rootstocks of both tobacco and thyme.

We then investigated whether grafting itself affected the transport of eugenol from the shoot to the root by grafting sweet basils. The quantity of eugenol in the shoots and roots of plants grafted between sweet basils was higher and lower, respectively, than that in non-grafted sweet basil. The eugenol content in the shoots of the sweet basil scion grafted onto tobacco or thyme rootstocks was comparable to that in the non-grafted sweet basil shoots (Figure 1I). In contrast, the quantity of eugenol in the roots of plants grafted between the sweet basil scion and tobacco or thyme rootstocks was almost half that in the non-grafted sweet basil (Figure 1J). However, the quantity was similar to that of eugenol in the roots of sweet basil-grafted plants. These results indicate that eugenol is transported from the shoots to the roots, even between different plant species.

### Eugenol could be synthesized in the root in addition to transporting it from the shoot

**Figure 2:**
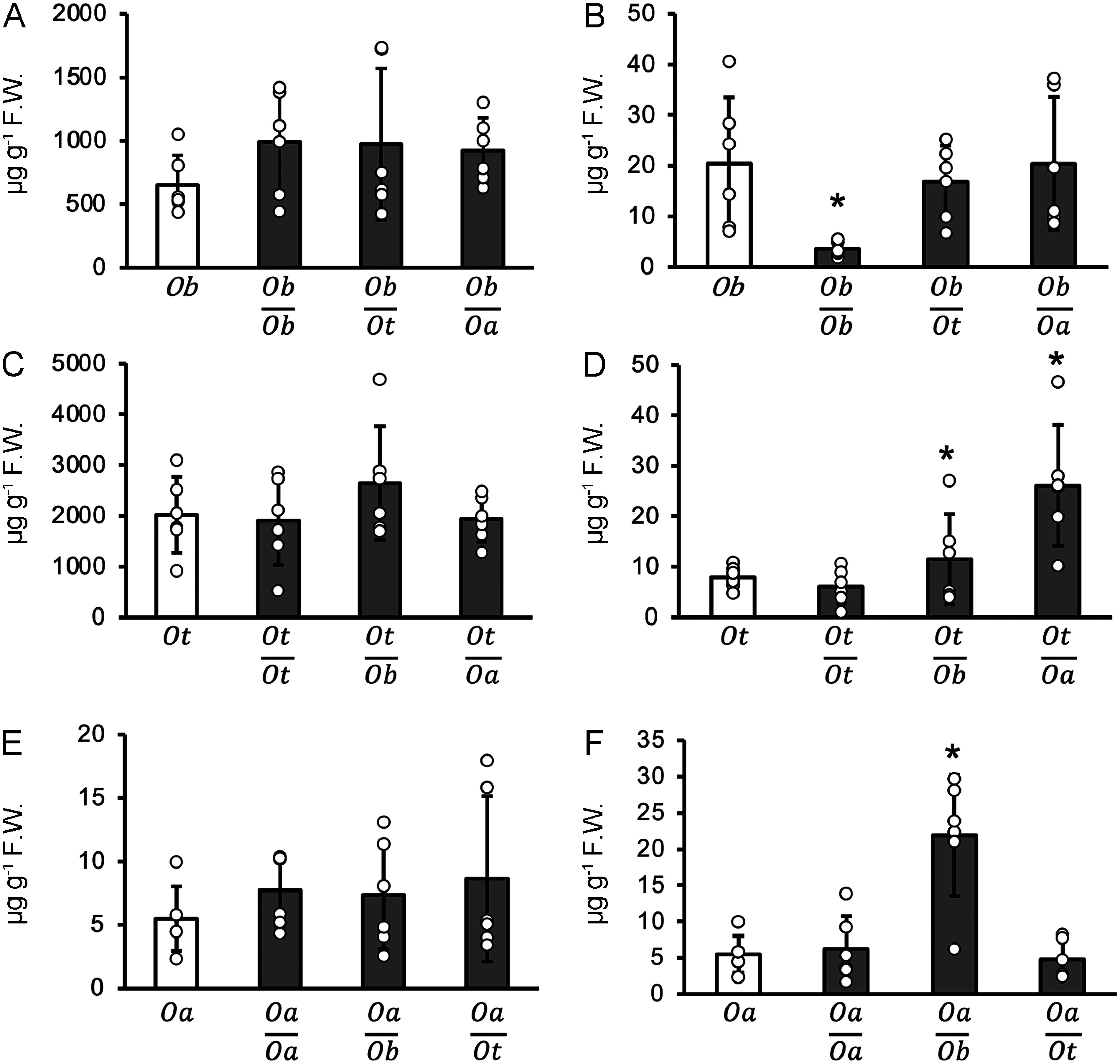
Figure 2. Eugenol quantity in grafted plants among the *Ocimum* genus. Comparison of eugenol levels in non-grafted and grafted plants among the *Ocimum* genus. (A) and (B) show the quantities of eugenol in the shoots and roots of non-grafted and grafted plants between the *O. basilicum* scion and *O. basilicum*, *O. tenuiflorum*, or *O. americanum* rootstocks, respectively. (C) and (D) show the quantities of eugenol in the shoots and roots of non-grafted and grafted plants between the *O. tenuiflorum* scion and *O. tenuiflorum*, *O. basilicum*, or *O. americanum* rootstocks, respectively. (E) and (F) show the quantities of eugenol in the shoots and roots of non-grafted and grafted between the *O. americanum* scion and *O. americanum*, *O. tenuiflorum*, or *O. basilicum* rootstocks, respectively. White boxes: non-grafted. Gray boxes: grafted. *Ob*; *O. basilicum*. *Ot*; *O. tenuiflorum*. *Oa*; *O. americanum*. Error bars represent±SD (*n*=6); * *p*<0.05 as determined by the Welch’s *t* test.

We found that eugenol was transported from the shoots to the roots of the grafted plants in distinct plant species. In support of our results, eugenol has been reported to be transported from the shoots to the roots through the phloem in *Ocimum kilimandscharicum* (Singh et al. 2020). To expand on this, we further investigated the quantity of eugenol transported within the *Ocimum* genus by grafting. Specifically, we performed grafting on three *Ocimum* species: *O*. *basilicum*, *O*. *tenuiflorum*, and *O*. *americanum*. The quantity of eugenol in the shoots of the *O*. *basilicum* scion did not change across all rootstock combinations (Figure 2A).

The quantity of eugenol was reduced in the roots of *O*. *basilicum* when grafted with *O*. *basilicum* as the scion (Figure 2B). The quantity of eugenol remained unchanged in the leaves of the *O*. *tenuiflorum* scion grafted across various rootstock combinations (Figure 2C). When *O*. *tenuiflorum* was the scion and either *O*. *basilicum* or *O*. *americanum* served as the rootstock, the quantity of eugenol increased in the roots (Figure 2D). Similar to the other combinations, the eugenol content in the shoots of the *O*. *americanum* scion remained unaltered across diverse rootstock combinations (Figure 2E). However, the quantity of eugenol in the roots increased when *O*. *americanum* was used as the scion and *O*. *basilicum* was used as the rootstock (Figure 2F).

Importantly, there was variability in the quantity of eugenol observed among the non-grafted members of *Ocimum*. *O*. *tenuiflorum* leaves exhibited the highest eugenol quantity, whereas *O*. *americanum* leaves exhibited considerably lower eugenol levels compared to the others. In contrast to the diverse eugenol quantities found in non-grafted leaves, the eugenol content in the roots of the non-grafted *Ocimum* remained relatively consistent. These results show that the quantity of eugenol in the shoots was not influenced by the choice of rootstock. However, fluctuations in eugenol content in the roots were dependent on the type of scion used.

### Eugenol was synthesized not only in the shoot but also in the root of *Ocimum*

Eugenol synthases (EGSs), the pivotal enzymes responsible for eugenol biosynthesis, have been identified in shoots. In the genus *Ocimum*, *EGS* exhibits specific expression and activity in PGTs, where eugenol accumulates (Koeduka et al. 2006; Lange and Turner 2013). In our grafting experiments, we observed variations in the eugenol content of the rootstocks, suggesting that eugenol is not only transported from the shoots, but also synthesized within the roots. We hypothesized that the changes in the quantity of eugenol that is synthesized in the roots might contribute to variations in the quantity of eugenol in the rootstocks. To ascertain the eugenol synthesis in the roots, we conducted an in vitro eugenol synthesis assay using crude protein extracts obtained from *Ocimum* roots.

**Figure 3:**
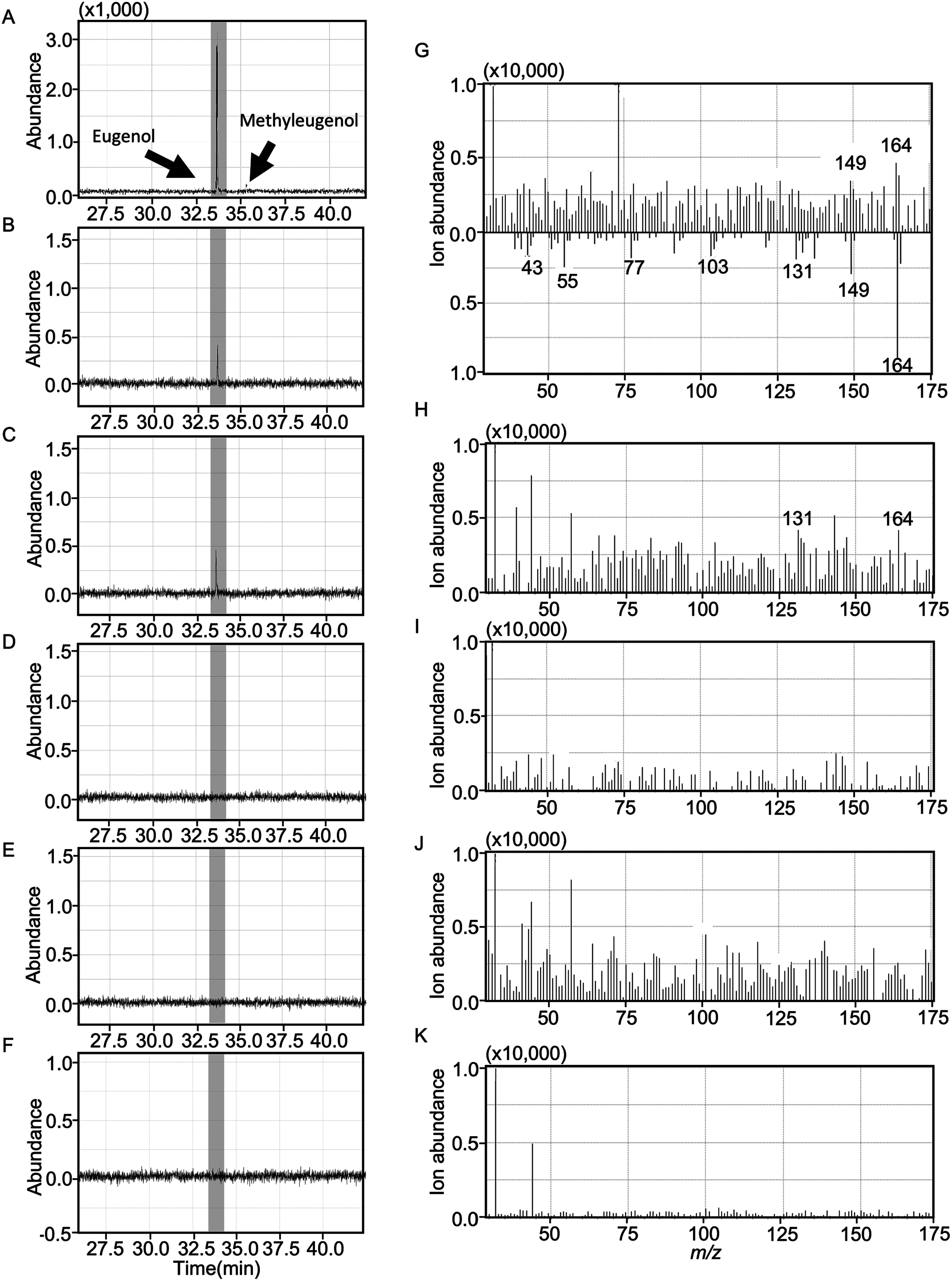
Figure 3. Eugenol biosynthetic activity in *O. basilicum* root. In vitro enzyme analysis of eugenol biosynthetic activity using the crude protein extract of *O. basilicum*. (A) to (F) depict the mass chromatograms of *m*/*z*=164 obtained using GC-MS. (A) Eugenol and methyleugenol standards. (B) Crude protein extracts from the *O. basilicum* shoot after 180 min of reaction with coniferyl alcohol. (C) Crude protein extracts from the *O. basilicum* roots after 180 min of the reaction with coniferyl alcohol. (D) Crude protein extracts from the *O. basilicum* roots after 180 min of the reaction without coniferyl alcohol. (E) Crude protein extracts from the *O. basilicum* roots after 0 min of the reaction with coniferyl alcohol. (F) Protein extraction buffer (without crude protein) after 180 min of incubation with coniferyl alcohol. (G) to (K) indicate the fragmentation patterns at approximately 33.7 min in (B) to (F), respectively. The bottom half of (G) shows the eugenol fragment pattern recorded in the Wiley Library (11th edition). (G) and (H) show eugenol-specific fragmentation patterns. Three biological replicates were performed, and representative results are shown.

To assess this assay, we reacted protein extracts obtained from the shoots of *O*. *basilicum*, *O*. *tenuiflorum*, and *O*. *americanum* with coniferyl alcohol, a substrate for EGSs. Subsequent GC-MS analysis detected eugenol-specific peaks in all the tested crude protein extracts (Figure 3B, G, and Supplementary Figures S1, S2). Conversely, eugenol was not detected in reaction mixtures lacking either protein extracts or coniferyl alcohol (Figure 3D, E, F, I, J, K). These results indicated that our enzyme assay effectively detected eugenol synthesized from coniferyl alcohol in vitro. In our in vitro assay utilizing protein extracts derived from shoots, the quantity of synthesized eugenol was the highest in *O*. *tenuiflorum* protein and the lowest in *O*. *americanum* protein (Supplementary Figure S3). This result is consistent with the results obtained from essential oil extraction from plant shoots, indicating that our in vitro assay accurately reflects the enzymatic activity of eugenol synthesis within the protein extracts.

In the root protein assay, we detected a eugenol peak in the GC-MS analysis using crude protein from *O*. *basilicum* roots (Figure 3C, H). This indicates that *O*. *basilicum* roots possess the ability to biosynthesize eugenol, albeit in low quantity. We also analyzed eugenol biosynthesis in crude protein extracts from the roots of *O*. *tenuiflorum* and *O*. *americanum*. These protein extracts also exhibited eugenol biosynthesis activity (Supplementary Figures S1, S2). These results strongly indicate that the examined members of the *Ocimum* genus possessed eugenol biosynthetic activity in their roots.

We further investigated the eugenol biosynthetic activity in the crude protein extract of *N*. *benthamiana* rootstock grafted with the *O. basilicum* scion. We determined whether enzymes involved in eugenol biosynthesis could be transported from the *O. basilicum* scion to *N*. *benthamiana* rootstocks. No eugenol peak was detected in the GC-MS analysis when using crude protein from *N*. *benthamiana* roots (Supplementary Figure S4). This result suggested that the presence of eugenol in *N*. *benthamiana* rootstocks grafted with the *O. basilicum* scion was not due to the transport of the enzyme proteins that involved in eugenol biosynthesis in *O. basilicum*.

### Grafting has small effect on the expression of *EGS* genes in *O. basilicum* root

Eight *EGS* genes have recently been identified in the *O*. *basilicum* genome (Reddy et al. 2021). Among them, *EGS1* was characterized as the gene with the highest expression in the shoot PGT of *O*. *basilicum*. *EGS4* exhibits the highest level of expression in the roots among the eight *EGS*s (Reddy et al. 2021). Because the sequences of *EGS*s have only been identified in *O*. *basilicum*, we investigated the expression of *EGS*s in both the scions and rootstocks of grafted *O*. *basilicum*. However, we encountered difficulties in designing specific primer sets that would allow for accurate quantification of the expression of *EGS2*, *EGS5*, and *EGS8* using qPCR, because of issues such as low PCR efficiency and detection of multiple PCR amplicons. Consequently, we quantified the expression levels of *EGS1*, *EGS3*, *EGS4*, *EGS6*, and *EGS7* using qPCR.

**Figure 4:**
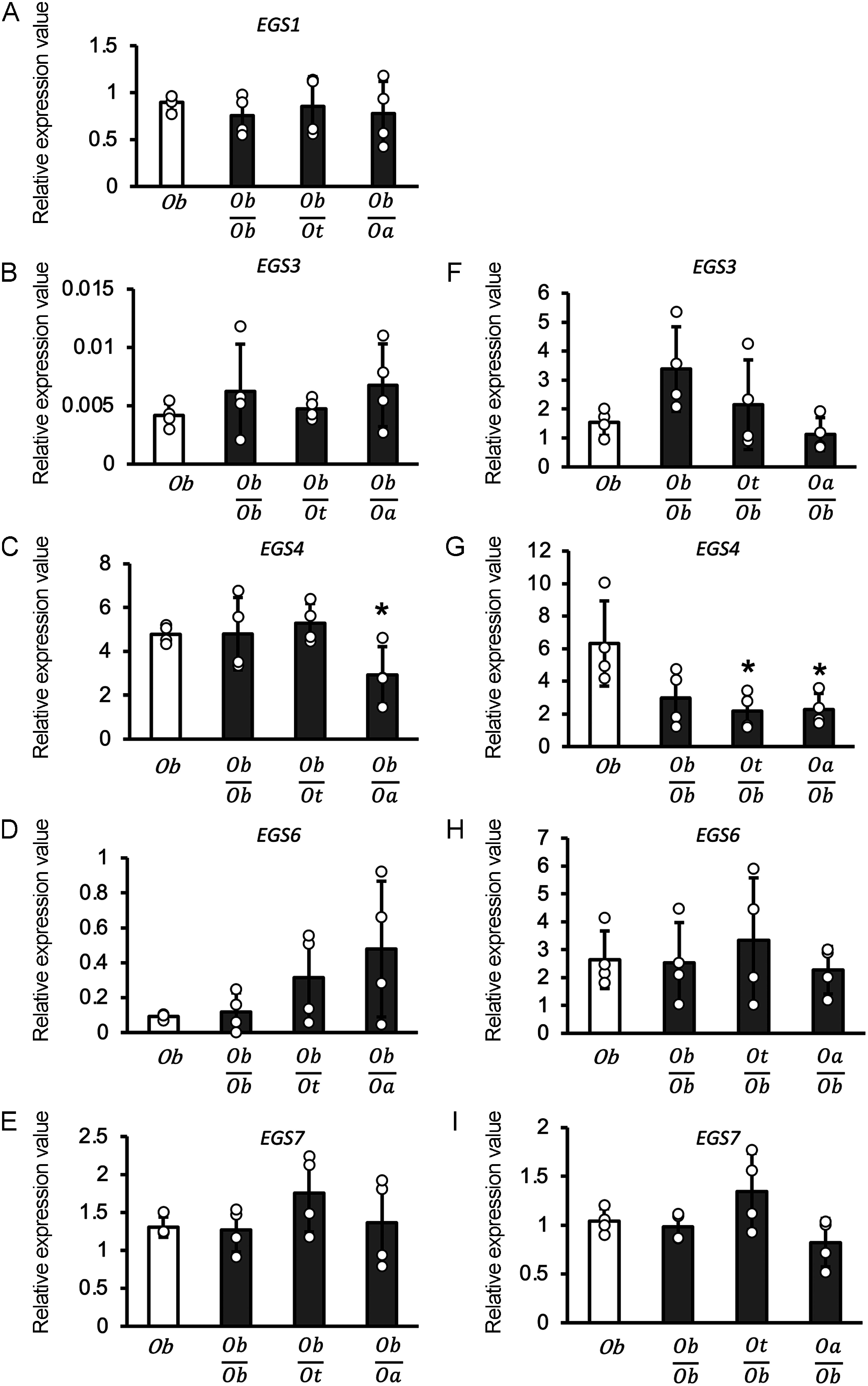
Figure 4. Expression analysis of *EGS* genes in non-grafted and grafted *O. basilicum*. (A) to (E) Expression analysis of eugenol synthesis genes (*EGS1*, *3*, *4*, *6*, and *7*) in leaves of non-grafted *O. basilicum* and plants grafted between *O. basilicum* scion and *O. basilicum*, *O. tenuiflorum*, and *O. americanum* rootstocks. (F) to (I) show the expression in the roots of non-grafted *O. basilicum* and plants grafted between *O. basilicum* rootstock and *O. basilicum*, *O. tenuiflorum*, and *O. americanum* scions. (A) *EGS1*, (B) and (F) *EGS3*, (C) and (G) *EGS4*, (D) and (H) *EGS6*, and (E) and (I) *EGS7*. *Ob*; *O. basilicum*. *Ot*; *O. tenuiflorum*. *Oa*; *O. americanum*. Each relative expression value was normalized to the level of β-*actin*. Error bars represent±SD (*n*=4); * *p*<0.05, as determined by the Student’s *t* test.

*EGS1* was expressed in shoots but not in roots (Figure 4A). *EGS3*, *EGS4*, *EGS6*, and *EGS7* were expressed in both the shoots and roots (Figure 4B, C, D, E, F, G, H, I). The expressions of *EGS1*, *EGS3*, and *EGS7* in the shoots were not altered regardless of grafting (Figure 4A, B, E). However, the expression of *EGS4* showed a significant reduction in grafts between *O*. *basilicum* scions and *O*. *americanum* rootstocks (Figure 4C). *EGS6* showed increased expression in the grafts of *O*. *basilicum* scions and *O*. *tenuiflorum* or *O*. *americanum* rootstocks, but the difference was not statistically significant (Figure 4D). The expression levels of *EGS3* and *EGS6* were higher in the roots than in shoots (Figure 4B, D, F, H). *EGS4* and *EGS7* exhibited similar expression levels in both the shoots and roots (Figure 4C, E, G, I). The expression levels of *EGS3*, *EGS6*, and *EGS7* in the roots did not change, regardless of grafting (Figure 4F, H, I). In contrast, the expression of *EGS4* in the roots significantly decreased after grafting, where *O*. *tenuiflorum* or *O*. *americanum* served as the scions and *O*. *basilicum* was used as the rootstock (Figure 4G). These results suggest that grafting had a minimal impact on the expression of *EGS* genes, except for *EGS4* in *O*. *basilicum* roots.

In the process of eugenol biosynthesis in *O. basilicum*, a crucial step involves the conversion of coniferyl alcohol from coniferyl acetate, which is the substrate of EGSs. This conversion is facilitated by coniferyl alcohol acetyltransferase (CFAT), marking a significant stage in the biosynthesis process. In *O. basilicum*, two *CFAT* genes were identified, namely *ObCAAT1* and *ObCAAT2* (Dhar et al. 2020). We also investigated *ObCAATs*’ expression in the grafted plants (Supplementary Figure S5). *ObCAAT1* expression was not affected by grafting. On the other hand, the expression level of *ObCAAT2* was higher in the *O. basilicum* scions grafted with rootstocks of *O. americanum* than that of the non-grafted *O. basilicum*. In contrast, the expression of *ObCAAT2* in the roots decreased after grafting, wherein *O*. *tenuiflorum* or *O*. *americanum* served as the scions and *O*. *basilicum* was used as the rootstock.

## Discussion

In the present study, we found that eugenol was transported from the shoots to the roots by grafting across various plant species, including sweet basil, tobacco, and thyme. Eugenol primarily undergoes biosynthesis in basil leaf PGTs and roots that grown in the soil (Reddy et al. 2021). Furthermore, eugenol has been shown to move from the shoots to the roots in *O*. *kilimandscharicum* (Singh et al. 2020). Our grafting experiments revealed that eugenol moved from shoots to roots, even between distinct species, as exemplified in this study, between sweet basil and tobacco and between sweet basil and thyme. Both our results and those of previous studies demonstrated that neither tobacco nor thyme originally contained eugenol (Courty et al. 2014; Paudel Timilsena et al. 2020). These results highlight the potential utility of eugenol in agricultural applications. Given that eugenol possesses antibacterial activity (Marchase et al. 2017), we can harness the antibacterial properties of eugenol from the basil scion by grafting it onto rootstocks incapable of eugenol biosynthesis.

Eugenol content not only varied between different species but also exhibited differences through grafting among the examined *Ocimum* genus. Interestingly, the eugenol content in shoots was not affected by grafting across all combinations of the examined *Ocimum* genus, namely, *O*. *basilicum*, *O*. *tenuiflorum*, and *O*. *americanum*. However, eugenol levels in the roots varied among the different grafting combinations of species of the genus *Ocimum*. Considering that the leaves of *O*. *tenuiflorum* exhibited the highest eugenol content among the tested leaves of *O*. *basilicum*, *O*. *tenuiflorum*, and *O*. *americanum*, it is plausible that the eugenol levels in the rootstocks of *O*. *basilicum* and *O*. *americanum* grafted with *O*. *tenuiflorum* as the scion were higher than those in non-grafted *O*. *tenuiflorum* or grafted with *O*. *tenuiflorum*. This suggests a potential link between the quantity of eugenol in the leaves and the transport of eugenol from shoots to roots.

We observed the movement of eugenol in the plants; however, the underlying molecular mechanisms remain to be elucidated. Previous studies have proposed several candidate genes that may play roles in eugenol transport. These include adenosine triphosphate-binding cassette transporters, multidrug and toxic compound extrusion efflux carriers, and sugars will eventually be exported transporters (SWEET) genes, which were selected through co-expression analysis with *EGS1* in *O*. *kilimandscharicum* (Singh et al. 2020). Le Roy et al. (2016) proposed that the long-distance transport of phenylpropanoids occurs through glycosylation and suggest the possible involvement of SWEET and similar proteins in the transportation of glycosylated eugenol in *O*. *kilimandscharicum*. The movement of eugenol in the grafted-plants may also occur in a glycosylated form. In the future, it will be important to examine the expression of these genes in grafted plants among other members of the *Ocimum* genus. Additionally, it is essential to determine the quantity of eugenol in grafted plants that have been genome-edited for these candidate genes and investigate whether the transported eugenol undergoes glycosylation or other modifications.

Furthermore, our results revealed the potential for eugenol biosynthesis in the roots through an in vitro enzyme assay. A previous study involving *O*. *kilimandscharicum* indicated the absence of *EGS1* expression in the root, suggesting that eugenol is not synthesized therein but rather transported from the shoot (Singh et al. 2020). However, different studies have shown that eugenol synthesis can occur in the roots of *O*. *basilicum* that is grown in soil or subjected to fungal infection or elicitor treatments (Reddy et al. 2021). Our results regarding the detection of eugenol in the roots can be attributed to the growth of grafted plants in the vermiculite. Notably, a previous study identified eight *EGS* genes through next-generation sequencing transcriptome analysis of *O. basilicum*, with *EGS1* showing no expression in the root. Among the *EGS* genes, *EGS4* showed the highest expression levels in roots. Moreover, the enzymatic activity of EGS4 is post-translationally regulated, particularly through protein phosphorylation (Reddy et al. 2021). Our results are consistent with *EGS4* expression in the roots of *O*. *basilicum*, even when the roots were subjected to grafting. A previous study showed that *EGS4* expression did not change between aseptic- and soil-grown basil (Reddy et al. 2021). Interestingly, we observed a significant reduction in the expression of *EGS4* in the grafted rootstocks of *O*. *basilicum*. This grafting-induced alteration in *EGS4* expression suggests its potential role in the regulation of transcription. Despite the decrease in *EGS4* expression observed in grafted roots, other *EGS* genes, such as *EGS3*, *EGS6*, and *EGS7* were expressed in the roots of *O*. *basilicum*. These EGSs are likely responsible for eugenol synthesis in *O*. *basilicum* roots in lieu of the reduced *EGS4* expression. This expression analysis was consistent with our in vitro enzyme assay, wherein crude protein extracts from *O*. *basilicum* roots displayed eugenol biosynthetic activity. Moreover, similar eugenol biosynthetic activities were observed in crude protein extracts from the roots of *O*. *tenuiflorum* and *O*. *americanum*. These results strongly suggest that the roots of *Ocimum* inherently possess eugenol biosynthetic capabilities that are distinct from their reliance on shoot-to-root transportation. However, the quantity of eugenol was reduced in the roots of *O*. *basilicum* grafted with *O*. *basilicum* as the scion, and even the *EGS3* expression in the grafted plants was higher than that in the non-grafted plants. We also investigated the expression of *ObCAAT1* and *2*, which are catalyzed one step before producing eugenol from coniferyl acetate. The activities of ObCAATs are also important for eugenol biosynthesis (Dhar et al. 2020). However, the expression levels of *ObCAAT1* and *2* were comparable between non-grafted *O*. *basilicum* and the *O*. *basilicum* scion grafted with the *O*. *basilicum* rootstock. These expression analyses suggest complex regulations of eugenol production, involving post-translational regulation of EGS proteins or chemical modification of eugenol itself. Moreover, it is possible that grafting might affect the transportation activity of eugenol. Elucidating the molecular mechanisms underlying eugenol biosynthesis in the roots and eugenol transportation from shoots to roots are of significant importance. If these mechanisms are fully understood, they can be harnessed to enhance antibacterial activity in the roots of *Ocimum* plants. By leveraging these mechanisms to stimulate eugenol synthesis, the potential enhancement of plant antibiotic properties could yield valuable benefits in agriculture.

In this study, we demonstrated that the *Ocimum* genus is endowed with the ability to transport eugenol from shoots to roots across various plant species. Moreover, we showed the inherent eugenol biosynthetic potential of *Ocimum* roots. These findings pave the way for future investigations into the molecular mechanisms governing eugenol transportation in the *Ocimum* genus or eugenol transport facilitated by grafting with the *Ocimum* genus. Owing to its potent antibacterial activity, eugenol has substantial commercial value in various industries, including medicine and food. Consequently, enhancing eugenol synthesis in roots and optimizing eugenol productivity through transportation, both utilizing plants, have significant societal implications.

## Abbreviations

CFAT: coniferyl alcohol acetyltransferase
EGS: eugenol synthase
GC: gas chromatography
GC-MS: gas chromatography-mass spectrometry
MES: 2-morpholinoethanesulfonic acid monohydrate
MS: Murashige and Skoog
PCR: polymerase chain reaction
PGT: peltate glandular trichomes

## Description of Supplementary Files

Supplementary Table S1. Primers used in this study.

Supplementary Table S2. Essential oil components identified in the shoot and root of *O*. *basilicum*.

Supplementary Figure S1. Eugenol biosynthetic activity in *O. tenuiflorum* root.

Supplementary Figure S2. Eugenol biosynthetic activity in *O. americanum* root.

Supplementary Figure S3. Eugenol quantity in the in vitro enzyme reaction mix with crude protein extracts from leaves of *O. basilicum*, *O. tenuiflorum*, and *O. americanum*.

Supplementary Figure S4. Eugenol biosynthetic activity in *N*. *benthamiana* root grafted with *O. basilicum* scion.

Supplementary Figure S5. Expression analysis of *ObCAAT1* and *ObCAAT2* genes in non-grafted and grafted *O. basilicum*.
