# Supplementary material for: Eugenol transport and biosynthesis through grafting in aromatic plants of the Ocimum genus
Source: Plant Biotechnol (Tokyo). 2024 Jun 25;41(2):111–20. doi: 10.5511/plantbiotechnology.24.0124a (PMC11500594; doi:10.5511/plantbiotechnology.24.0124a)
Supplement: Supplementary Data [file plantbiotechnology-41-2-24.0124a-s001.pdf]

Supplementary Files

Eugenol transport and biosynthesis through grafting in aromatic plants of the *Ocimum* genus.

Shogo Hirose, Kaito Sakai, Sawa Kobayashi, Masato Tsuro, Atsushi Morikami, Hironaka Tsukagoshi*

*Corresponding Author: thiro@meijo-u.ac.jp

This PDF file includes:

Supplementary Table S1 and S2

Supplementary Figure S1 to S5

Supplementary references

Supplementary Table S1. Primers used in this study

Primer name	Sequence(5' to 3')	Source
Ob_actin_Fw	GCACTTTTCCTGTGAACAATAG	(Rastogi et al., 2014)
Ob_actin_Rv	AGAGGATACATGTTCACCAC	
q_EGS1-Fw	GGTGCCATCATAGTCAAGGG	(Reddy et al., 2021)
q_EGS1-Rv	GCAATGCGTTTATTCTGTCCT	
q_EGS3-Fw	CGTCGGAGTTTGGTTGTGAT	
q_EGS3-Rv	GAGGCTCAGTTCTATTAGGTTGC	
q_EGS4-Fw	CGCGCTGCAGTTGGGAGATC	
q_EGS4-Rv	AGAGGCCAGGCTGGGACAG	
q_EGS6-Fw	CAACGACGAACGCGACATTGGGGCATAC	
q_EGS6-Rv	CACCGGAATCGGCGACTCTTGG	
q_EGS7-Fw	GACCGTGTCCATGCTGTTGAGCCG	
q_EGS7-Rv	TGGGGTAACTGTGGCGTCTAACTGA	
q_CAAT1-Fw	AAGCTCGACCTCTCCCAACC	This study
q_CAAT1-Rv	GCGACTGGAATGTGGAGAAG	
q_CAAT2-Fw	AGGAATATAATCCAGCTGTCGG	(Dhar et al., 2020)
q_CAAT2-Rv	AGGAACAAGTCTGGCATGAACAGA	

Supplementary Table S2. Essential oil components identified in the shoot and root of *O. basilicum*

A

No.	RT	compound	Relative percentage (%)
1	8.287	α-Pinene	0.0334 ± 0.0003
2	11.267	β-Pinene	0.0011 ± 0.0005
3	12.500	Myrcene	0.0006 ± 0.0004
4	16.024	Limonene	0.0022 ± 0.0014
5	16.377	1,8-cineole	1.5071 ± 0.5512
6	18.833	Ocimene	0.0896 ± 0.0676
7	24.541	Linalool	2.2645 ± 2.3439
8	27.215	Camphor	0.0672 ± 0.0484
9	27.289	Borneol	0.0982 ± 0.0283
10	29.852	α-Terpineole	0.1587 ± 0.0805
11	36.288	Eugenol	7.2089 ± 1.9036
12	38.012	Tetradecane	26.0500 ± 2.5482
13	38.141	Methyl Eugenol	0.0832 ± 0.0252

B

No.	RT	compound	Relative percentage (%)
1	36.237	Eugenol	0.3240 ± 0.0020
2	38.812	Tetradecane	28.3552 ± 7.9234

Chemicals detected in *O. basilicum* shoots and roots using GC analysis. RT; retention time. Tetradecan was added to the essential oils as an internal standard for the GC analysis. The relative percentages of essential oils were calculated by dividing each peak by the total area acquired from the GC analysis. n = 6, ± SD.

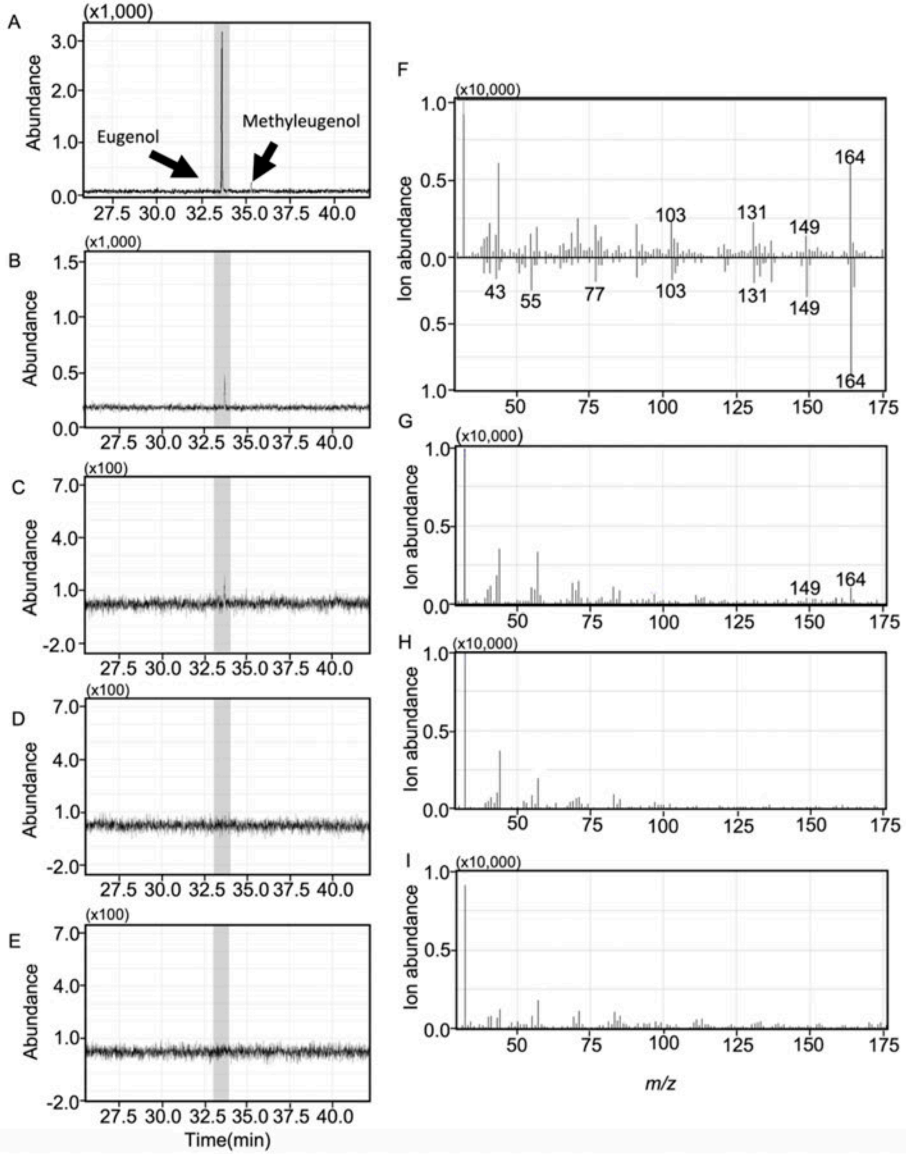
A
(x1,000)
Abundance
Eugenol
Methyleugenol
27.5 30.0 32.5 35.0 37.5 40.0
B
(x1,000)
C
(x100)
D
(x100)
E
(x100)
Time(min)
F
(x10,000)
Ion abundance
164
103
131
149
43
55
77
G
(x10,000)
H
(x10,000)
I
(x10,000)
m/z

Supplementary Figure S1. Eugenol biosynthetic activity in *O. tenuiflorum* root.

In vitro enzyme analysis of eugenol biosynthesis using crude protein extracts of *O. tenuiflorum*. (A) to (E) depict the mass chromatograms of m/z = 164 obtained using GC-MS. (A) Eugenol and methyleugenol standards. (B) Crude protein extracts from the *O. tenuiflorum* shoots after 180 min of reaction with coniferyl alcohol. (C) Crude protein extracts of *O. tenuiflorum* roots after 180 min of reaction with coniferyl alcohol. (D) Crude protein extracts from the *O. tenuiflorum* roots after 180 min of reaction without coniferyl alcohol. (E) Crude protein extracts from *O. tenuiflorum* roots after 0 min of reaction with coniferyl alcohol. (F) to (I) Fragmentation patterns at approximately 33.7 min in (B) to (E), respectively. The bottom half of (F) shows the eugenol fragment pattern recorded in the Wiley Library (11th edition). (F) and (G) show eugenol-specific fragmentation patterns. Three biological replicates were performed, and representative results are shown.

.

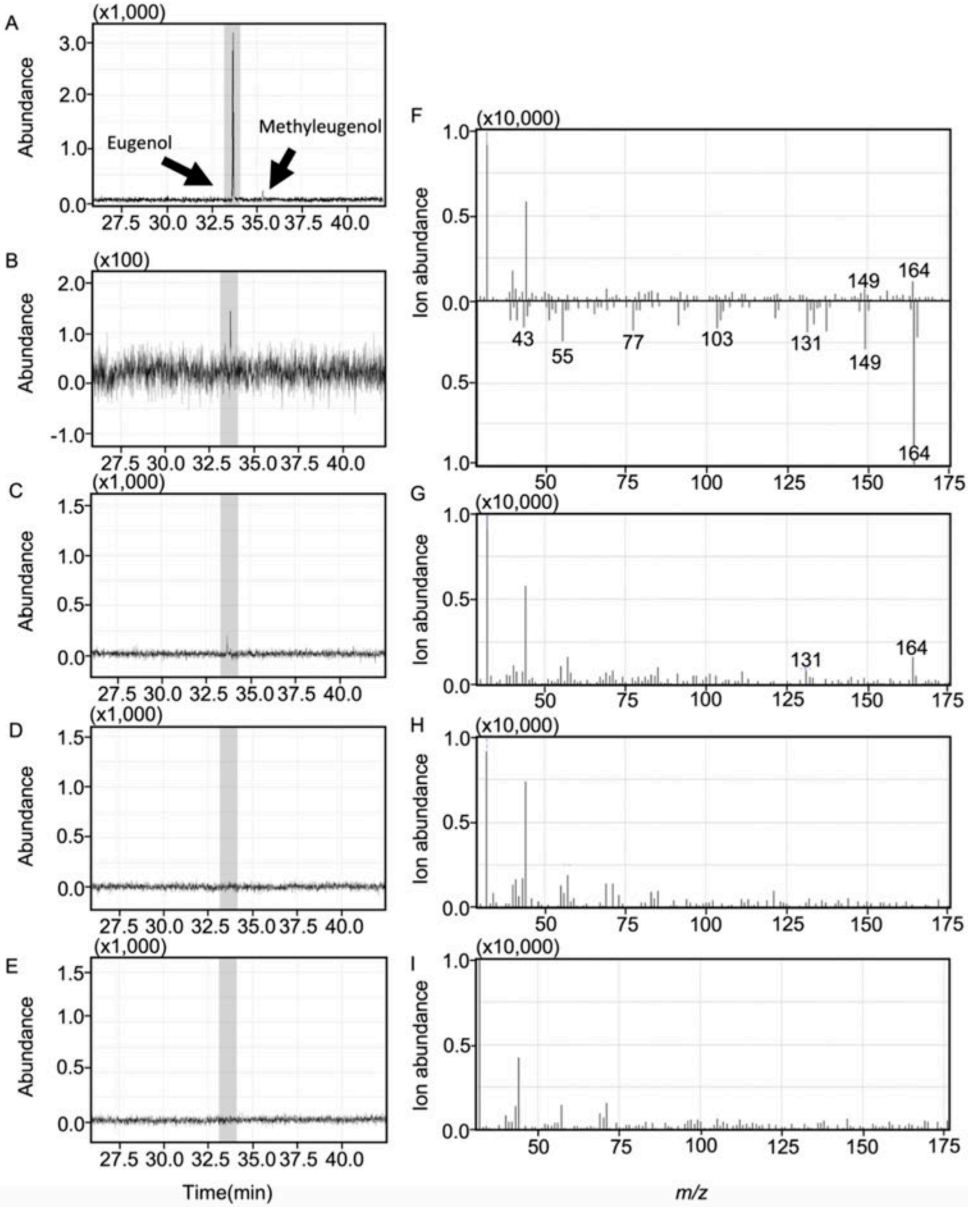
A
B
C
D
E
F
G
H
I
(x1,000)
(x100)
(x10,000)
Abundance
Ion abundance
Eugenol
Methyleugenol
43
55
77
103
131
149
164
Time(min)
m/z

Supplementary Figure S2. Eugenol biosynthetic activity in *O. americanum* root.

In vitro enzyme analysis of eugenol biosynthesis using crude protein extracts of *O. americanum*. (A) to (E) depict the mass chromatograms of *m/z* = 164 obtained using GC-MS. (A) Eugenol and methyleugenol standards. (B) Crude protein extracts from the *O. americanum* shoots after 180 min of reaction with coniferyl alcohol. (C) Crude protein extracts of *O. americanum* roots after 180 min of reaction with coniferyl alcohol. (D) Crude protein extracts of the *O. americanum* roots after 180 min of reaction without coniferyl alcohol. (E) Crude protein extracts of *O. americanum* roots after 0 min of reaction with coniferyl alcohol. (F) to (I) Fragmentation patterns at approximately 33.7 min in (B) to (E), respectively. The bottom half of (F) shows the eugenol fragment pattern recorded in the Wiley Library (11th edition). (F) and (G) show eugenol-specific fragmentation patterns. Three biological replicates were performed, and representative results are shown.

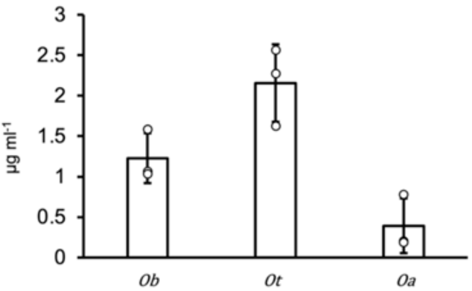

Supplementary Figure S3. Eugenol quantity in the *in vitro* enzyme reaction mix with crude protein extracts from leaves of *O. basilicum*, *O. tenuiflorum*, and *O. americanum*.

Eugenol content in *in vitro* enzyme reaction mix using crude protein extracts from leaves of *O. basilicum*, *O. tenuiflorum*, and *O. americanum*. (GC data are shown in Figure 3B, Supplementary Figure 1B, Supplementary Figure 2B), Error bars represent ± SD (n=3).

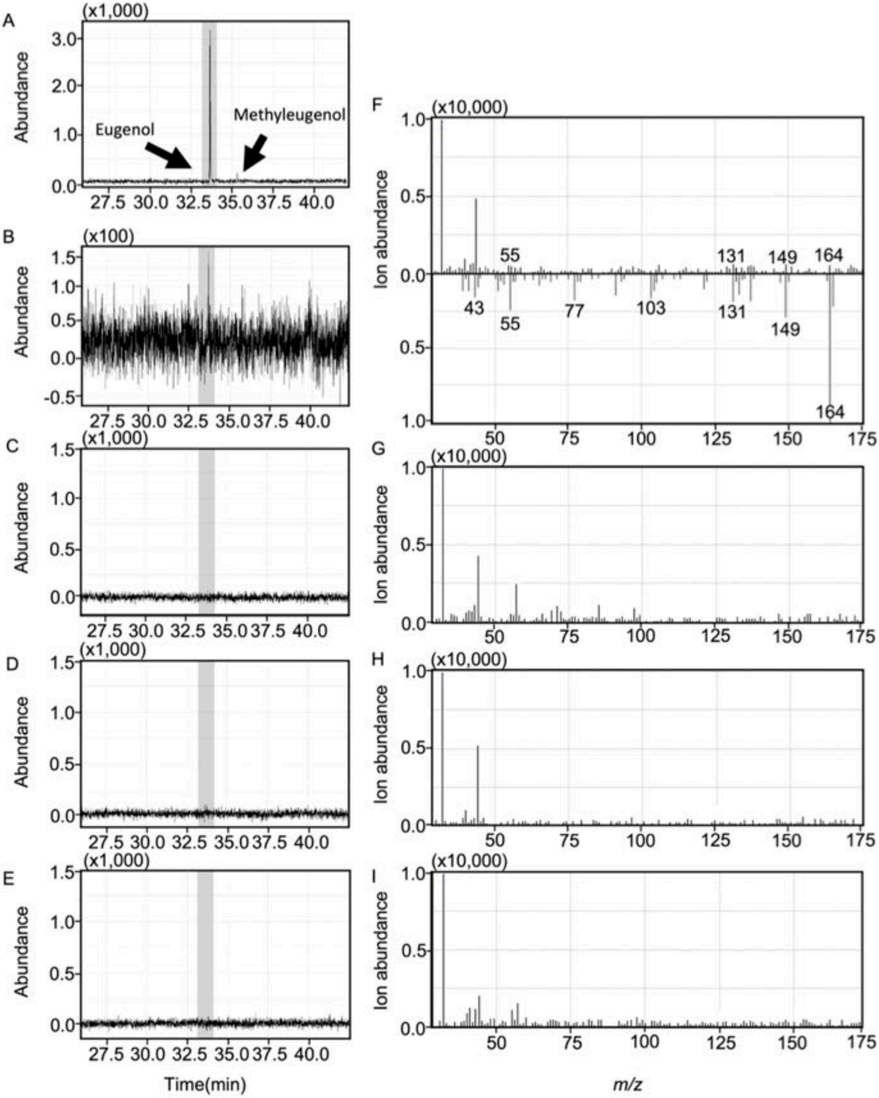
A
B
C
D
E
F
G
H
I
(x1,000)
(x100)
(x10,000)
Abundance
Ion abundance
Eugenol
Methyleugenol
43
55
77
103
131
149
164
Time(min)
m/z

Supplementary Figure S4. Eugenol biosynthetic activity in *N. benthamiana* root grafted with *O. basilicum* scion.

In vitro enzyme analysis of eugenol biosynthesis using crude protein extracts of *N. benthamiana* rootstocks grafted with *O. basilicum* scion. (A) to (E) depict the mass chromatograms of *m/z* = 164 obtained using GC-MS. (A) Eugenol and methyleugenol standards. (B) Crude protein extracts from the scion of *O. basilicum* after 180 min of reaction with coniferyl alcohol. (C) Crude protein extracts of *N. benthamiana* roots after 180 min of reaction with coniferyl alcohol. (D) Crude protein extracts of the *N. benthamiana* roots after 180 min of reaction without coniferyl alcohol. (E) Crude protein extracts of *N. benthamiana* roots after 0 min of reaction with coniferyl alcohol. (F) to (I) Fragmentation patterns at approximately 33.7 min in (B) to (E), respectively. The bottom half of (F) shows the eugenol fragment pattern recorded in the Wiley Library (11th edition). (F) and (G) show eugenol-specific fragmentation patterns. Three biological replicates were performed, and representative results are shown.

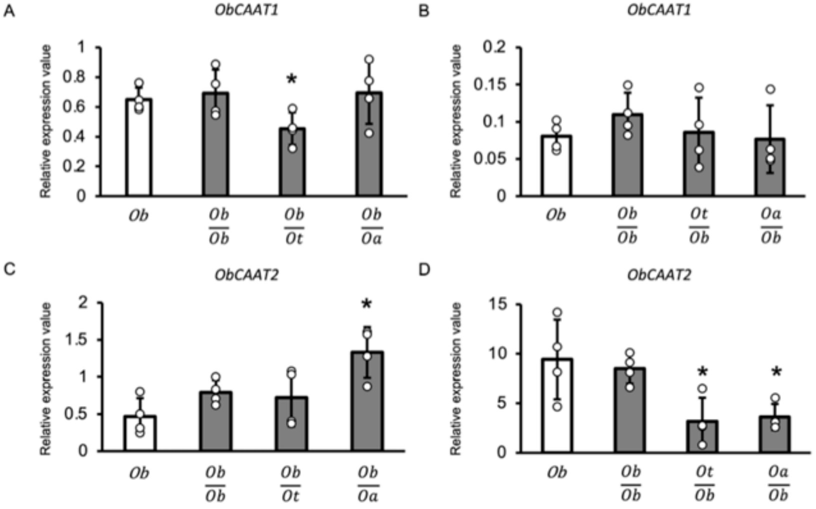

Supplementary Figure S5. Expression analysis of *ObCAAT1* and *ObCAAT2* genes in non-grafted and grafted *O. basilicum*.

(A) and (C) Expression analysis of coniferyl alcohol acetyltransferases (*ObCAAT1* and *ObCAAT2*) in leaves of non-grafted *O. basilicum* and plants grafted between *O. basilicum* scion and *O. basilicum*, *O. tenuiflorum*, and *O. americanum* rootstocks. (B) and (D) show the expression in the roots of non-grafted *O. basilicum* and plant grafted between *O. basilicum* rootstock and *O. basilicum*, *O. tenuiflorum*, and *O. americanum* scions. (A) and (B) *ObCAAT1* and (C) and (D) *ObCAAT2*. *Ob*; *O. basilicum*. *Ot*; *O. tenuiflorum*. *Oa*; *O. americanum*. Each relative expression value was normalized to the level of *β-actin*. Error bars represent ± SD (n = 4); * $p < 0.05$, as determined by the Student's *t* test.

Supplementary references

Dhar N, Saranagapani S, Reddy VA, Kumar N, Panicker D, Jin J, Chua N-H, Sarojam R (2020) Characterization of a sweet basil acyltransferase involved in eugenol biosynthesis. *J Exp Bot* 71: 3638-3652

Rastogi S, Mena S, Bhattacharya A, Ghosh S, Shukla RK, Sangwan NS, Lal RK, Gupta MM, Lavania UC, Gupta V et al. (2014) De novo sequencing and comparative analysis of holy and sweet basil transcriptomes. *BMC genomics* 15: 588

Reddy VA, Li C, Nadimuthu K, Tjhang JG, Jang IC, Rajani S (2021) Sweet basil has distinct synthases for eugenol biosynthesis in glandular trichomes and roots with different regulatory mechanisms. *Int J Mol Sci* 22: 681
